# Lignin Structure and Solvent Effects on the Selective Removal of Condensed Units and Enrichment of S-Type Lignin

**DOI:** 10.3390/polym10090967

**Published:** 2018-09-01

**Authors:** Si Gao, Ji Zhao, Xing Wang, Yanzhu Guo, Ying Han, Jinghui Zhou

**Affiliations:** 1 Liaoning Key Laboratory of Pulp and Papermaking Engineering, Dalian Polytechnic University, Dalian 116034, China; gsdalian@163.com (S.G.); guoyz@dlpu.edu.cn (Y.G.); hanying@dlpu.edu.cn (Y.H.); 2 Jining Ming Sheng New Material Co., Ltd., Jinan 272100, China; zhaoji@dssun.com; 3 State Key Laboratory of Pulp and Papermaking Engineering, South China University of Technology, Guangzhou 510640, China

**Keywords:** lignin purification, structural recognition, condensed lignin, S/G ratio

## Abstract

This study focused on the structural differences of lignin after pyridine–acetic acid–water (PAW) and dioxane–acidic water (DAW) purification processes. These structural differences included the S/G ratio, condensed structure, weight-average (M_W_) molecular weights, β-O-4 linkages and sugar content. The chemical structure of the isolated crude lignin (CL), PAW purified lignin (PPL) and DAW purified lignin (DPL) was elucidated using quantitative ^13^C NMR, 2D-HSQC NMR spectra, thermogravimetric analysis (TGA), gel permeation chromatography (GPC) and Fourier transform infrared spectroscopy (FTIR). The results showed that the PPL fractions contain fewer condensed structures, higher S/G ratios, more β-O-4 linkages, higher average M_W_ and lower thermal degradation properties compared to the CL and DPL fractions. Furthermore, the PAW process was more selective in removing condensed units and enriching S-type lignin from CL compared to the DAW process. These results provide valuable information for understanding which purification process is more suitable to be applied for lignin.

## 1. Introduction

Lignin, which is a dominant aromatic polymer found in nature, is found in most terrestrial plants at a concentration of approximately 15%–40% of the dry weight and provides structural integrity [1]. Research and development activities that have been directed towards the commercial production of cellulosic ethanol have created an opportunity to dramatically increase the transformation of lignin to value-added products, including low-cost carbon fibers, engineered plastics and thermoplastic elastomers, polymeric foams, fungible fuels and commodity chemicals [1,2,3].

It is well-known that lignin has an amorphous and complex structure. Furthermore, its structure and properties change according to certain reactions during the biomass pretreatment processes. Therefore, the valorization of lignin is highly restricted by its heterogeneous features in terms of structural diversity, high polydispersity, non-uniformed reactivity, low reactivity of condensed lignin [4], wide chemical group composition [5] and so on. The structure of lignin could considerably change based on the sources of feedstock, process conditions and the isolation method, which would lead to different physicochemical properties. For instance, in the auto-catalyzed ethanol organosolv pretreatment process, the produced lignin tends to have high purity, low molecular mass, high phenolic and carboxyl contents and a condensed G-type structure, while it is free of sulfur and ash [6]. The diversity of various functional groups that are present in auto-catalyzed ethanol organosolv lignin makes its utilization quite challenging. In this respect, many purification methods have been proposed for the production of more uniformed lignin. The three main methods are related to the extraction of lignin with different organic solvents [7,8,9,10], ultrafiltration by membrane technology [11,12] and fractionation by acid precipitation [5,13]. An early work by Lundquist et al. [14] focused on the isolation of lignin using pyridine–acetic acid–water (PAW) organic solvents and had characterized the properties of purified lignin. A similar study was also conducted by Yang et al. [15] using dioxane–acidic water (DAW) organic solvents to isolate lignin from wheat straw and obtained fractions, which were subsequently chemically and thermally characterized. As the recent advances in the valorization of lignin, the PAW and DAW processes are usually used as the purification methods for lignin samples. Hu et al. [16] used the DAW process to purify enzymatic/mild acidolysis lignin, before evaluating the inhibitory effects of pseudolignin on the enzymatic hydrolysis of cellulose compared to lignin. In similar studies, Li et al. [17] and Lv et al. [18] also adopted this purification method. However, in other studies examining lignin application, the PAW process has also been widely used. Before the utilization of sorghum lignin to improve the adhesive strength of soy protein adhesives, Xiao et al. [19] used the PAW process to successfully purify sorghum lignin. Dong et al. [20], Tian et al. [21] and Sadeghifar et al. [22] also adopted this PAW process to isolate the lignin. However, the structural differences of lignins after the DAW and PAW purification processes have not been investigated. The knowledge of the structural changes of lignin due to the purification processes can provide valuable information for understanding which purification process was more suitable to be subsequently applied for lignin.

For this reason, the structural differences of lignin after the DAW and PAW purification processes, such as S/G ratio, condensed structure, M_W_, β-O-4 linkages and sugar content, was extensively investigated in this present work. The characterization of the correspondingly produced lignin was carried out by the means of quantitative ^13^C NMR, 2D-HSQC NMR spectra, thermogravimetric analysis (TGA), gel permeation chromatography (GPC), Fourier transform infrared spectroscopy (FTIR) and ionic chromatography.

## 2. Materials and Methods 

**Materials.** Poplar wood chips (dimensions: 4 cm × 4 cm × 2 cm) were collected from Tiger Forest & Paper Co., Ltd. (Hunan, China). The moisture content in chips was determined to be 15.4 ± 0.2 wt %. All chemical reagents used in this paper were purchased from Sigma-Aldrich (Darmstadt, Germany), Aladdin (Shanghai, China) and Sinopharm Chemical Reagent Co., Ltd. (SCRC) (Beijing, China). 

**Crude lignin extraction.** Crude lignin was extracted from the poplar wood chips by the organosolv technique using ethanol/water as solvents according to our previous report [23]. A mixture of 80 g of poplar wood in 800 mL of ethanol/water (*v*/*v*, 60%) was stirred at 205 °C for 2 h. The mixture was cooled, filtered and washed with solvents (ethanol/water). The ethanol–water soluble portion was concentrated to 200 mL at 50 °C under reduced pressure in a rotary evaporator (RV10 control, IKA Company, Staufen, Germany), before being poured into 600 mL of distilled water to precipitate lignin. The precipitates were collected by membrane filtration and washed with acidic water (pH of 2.0) five times. After freeze drying, a dark brown crude lignin was obtained, which was named as CL.

**Dioxane/acidified water purified lignin.** The CL isolated from the poplar wood chips was purified through the dioxane/acidified water (85:15 *v*/*v*) (DAW) process [15]. Five grams of crude lignin was suspended in 100 mL of dioxane–acidic water solution (0.01 mol/L HCl). After this, the mixture was refluxed at 85 °C in a nitrogenous environment for 2 h. The lignin was subsequently filtered and washed with dioxane-acidic water (85:15 *v*/*v*). The filtrate was evaporated in a rotary evaporator at 40 °C, before the lignin was precipitated in water. The precipitated lignin was subsequently centrifuged and freeze-dried. After freeze-drying, the obtained lignin was named as DPL.

**Pyridine–acetic acid–water purified lignin.** The CL isolated from the poplar wood chips was purified through the pyridine-acetic acid-water (9:1:4) (PAW) process [8]. Five grams of crude lignin was suspended in 140 mL of pyridine–acetic acid–water solution. The solution was mixed with 150 mL of chloroform, before being centrifuged to separate the aqueous and non-aqueous layers. The non-aqueous layer was isolated and the solvent was removed by evaporation. The lignin was redissolved in 10 mL of 1,2-dichloroethane/ethanol (2:1, *v*/*v*) and precipitated by the dropwise addition into 250 mL of diethyl ether. The precipitated lignin was washed twice with ether and air-dried, before the obtained lignin was named as PPL.

**Analytical Methods.** The chemical compositions of CL, DPL and PPL, including the amounts of sugars and lignin, were determined according to the National Renewable Energy Laboratory standard analytical method (NREL/TP-510-42618) [24]. Every experiment was repeated two times. The sugar contents of CL, DPL and PPL samples were determined by ionic chromatograph (Dionex ICS-5000, Thermo Scientific, Waltham, MA. USA), which was equipped with a Capillary Reagent-Free IC System (Thermo Scientific, Waltham, MA, USA) and a CarboPac 20 column (Thermo Scientific, Waltham, MA, USA).

The FTIR spectra of lignin-rich residues were obtained using a JASCO FT/IR-460 Plus spectrometer (JASCO, Tokyo, Japan), which was equipped with an accessory single reflection diamond and operated for 400 scans with a resolution of 1 cm^−1^ and a spectral range of 4000–600 cm^−1^. The FTIR bands were determined by comparing them with those reported in the literature [25].

The NMR determinations for all of the prepared lignin samples were performed in a Bruker AVIII 400 MHz spectrometer (Bruker Daltonic Inc., Bremen, Germany). For the ^13^C NMR analysis, 140 mg of lignin samples were dissolved in 0.5 mL of DMSO-*d*_6_. The ^13^C NMR spectra were acquired in the FT mode at 100.6 MHz by employing an inverse-gated decoupling pulse sequence, which was conditioned with the following parameters: a pulse angle of 30°, pulse delay of 2 s, acquisition time of 1.4 s and 30,000 scans at room temperature. The signals in the ^13^C NMR spectra were assigned according to the previous literature [26]. The 2D-HSQC NMR determination was carried out according to the recently reported method [23,26] with minor modifications. Briefly, 140 mg of lignin samples were dissolved in 0.5 mL of DMSO-*d*_6_. The standard pulse program hsqcedetgpsisp2 was applied in the HSQC experiment. The spectral widths for the ^13^C and ^1^H dimensions were 20,000 Hz and 5000 Hz, respectively. The central solvent peak was used as an internal reference (δ_C_ 39.5; δ_H_ 2.49).

The molecular weights of lignin preparations were analyzed by Gel Permeation Chromatography (GPC) (Waters, Milford, MA, USA), which was based on the procedures described in a previous study [27]. The analysis of the thermal stability of lignin samples was conducted on a TA Q500 thermal analyzer (TA Instruments, New Castle, DE, USA), which was processed by heating the samples from 30 to 600 °C at a heating rate of 10 °C min^−1^ under the protection of nitrogen.

## 3. Results and Discussion

### 3.1. Yield and Composition of Lignin Fractions

**Figure 1:**
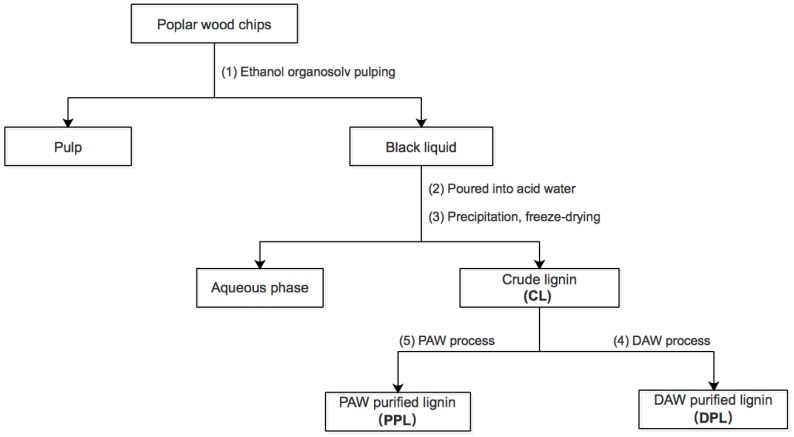
Sequential lignin samples.

The purity of the crude lignin (CL) sample was based on the DAW and PAW processes (Figure 1), with the lignin yields and the chemical compositions given in Table 1. The yield of DPL with the DAW process from CL was 77.3%, while the yield of PPL with the PAW process was 43.9%. It could be concluded that the PAW process was more selective for certain structures of lignin compared to the DAW process. The contents of total sugars in these three lignin fractions were 2.11%, 0.82% and 0.21%, respectively. This indicated that the removal effect of the PAW process was higher than that of the DAW process. Additionally, xylan was the major sugar in CL, DPL and PPL. Furthermore, the content of xylan in DPL and PPL was lower than that in CL, which implies that the hemicelluloses attached to lignin were partially removed during the DAW and PAW processes. Meanwhile, the PAW process exhibited a higher selective removal of lignin that contained hemicellulose from CL.

### 3.2. Internal Linkages and Aromatic Units

**Figure 2:**
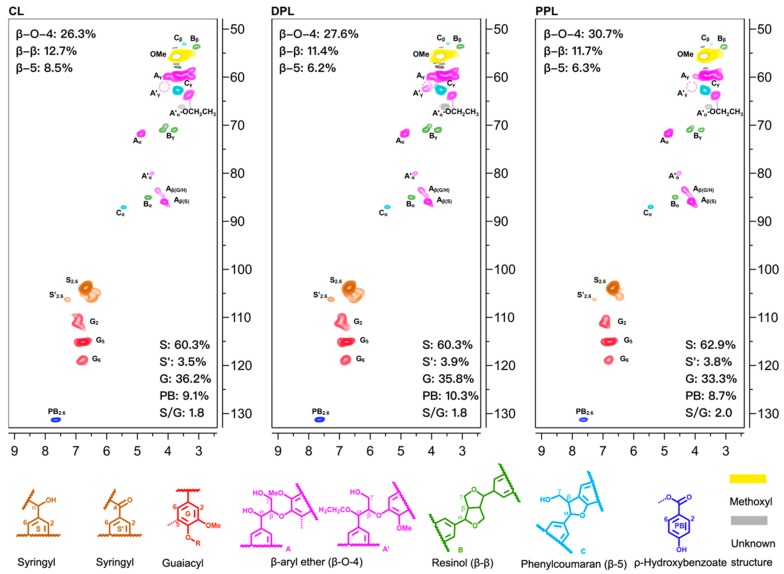
Partial 2D-HSQC NMR spectra of CL, DPL and PPL. Volume integrals are given for the lignin side-chain structures, which are color-coded to match their signal assignments in the spectrum.

The 2D-HSQC NMR method, which has been widely used for elucidating the lignin structure [28], was employed for the characterization of CL, DPL and PPL. Figure 2 compares the NMR spectra of the parent crude lignin and its purified forms during the DAW and PAW processes. This 2D-HSQC NMR spectrum consists of two regions: the oxygenated region (δ_C_/δ_H_ 50–90/2.5–5.7 ppm) and the aromatic region (δ_C_/δ_H_ 100–135/6.0–8.0 ppm), which are shown in Figure 2. Based on the previous studies [26], the main signals were assigned and the detected linkages are depicted in Figure 2, such as the inter-unit linkages β-O-4 aryl ether (A), resinol (β–β, B) and phenylcoumaran (β-5, C). The cross-signals of C_α_–H_α_ in the β-O-4 substructure are explicitly located at δ_C_/δ_H_ 71.8/4.86, while the cross-signals at δ_C_/δ_H_ 84.3/4.30 and 86.5/4.12 are attributed to its C_β_–H_β_ correlations, which are linked to the G and S units in β-O-4 substructures, respectively. Meanwhile, the resinol (β–β, B) was also detected, while the C_α_–H_α_, C_β_–H_β_ and C_γ_–H_γ_ correlations were observed at δ_C_/δ_H_ 85.5/4.67, 54.1/3.08, 71.6/4.21 and 3.85, respectively. Moreover, the phenylcoumaran (β-5, C) was found at C_α_–H_α_ (δ_C_/δ_H_ 87.1/5.44), C_β_–H_β_ (δ_C_/δ_H_ 54.1/3.73) and C_γ_–H_γ_ (δ_C_/δ_H_ 62.9/3.72). In addition, the signals for the Cα-oxidized S units (δ_C_/δ_H_ 62.9/3.72) and the signals for the Cα-oxidized S units (S’) was also observed at δ_C_/δ_H_ 64.5/3.35 [29].

The influence of the organic solution on the structural features during the DAW and PAW processes were analyzed by the comparison of the 2D-HSQC NMR spectra of CL, DPL and PPL samples. In the aliphatic side-chain regions, the signals at δ_C_/δ_H_ 64.4/3.35 and 80.6/4.55 were observed in the three spectra, which were assigned to the methylene groups in α-ethoxylated β-O-4 linkages and the α-position of α-acylated β-O-4 linkages, respectively. This indicates that the hydroxyl groups in the α-position of β-O-4 linkages (A) were possibly replaced by the nucleophilic ethanol co-solvent, which finally generated the α-ethoxylated β-O-4 linkages (A’) during ethanol organosolv pulping [6,26]. The cross-signals of C_γ_–H_γ_ (δ_C_/δ_H_ 62.8/4.17) in the α-ethoxylated β-O-4 linkages (A’) [23,26] were not found in the spectra of CL and PPL samples but they were observed in DPL. This indicated that the DAW process was more selective for the α-ethoxylated β-O-4 linkages (A’) structures compared to the PAW process. In the aromatic region of the HSQC spectra, the signals from syringyl (S), guaiacyl (G) and *p*-hydroxyphenyl (H) units were distinguished. As shown in Figure 2 (CL), the S units were observed as a prominent signal in CL for the C_2,6_–H_2,6_ correlation at around δ_C_/δ_H_ 104.6/6.77, while the signals for Cα-oxidized S units (S’) were observed at δ_C_/δ_H_ 106.3/7.20 ppm. G units were also observed with the correlations for C_2_–H_2_ at δ_C_/δ_H_ 109.9/7.22 ppm, C_5_-H_5_ at δ_C_/δ_H_ 115.8/6.81 ppm and C_6_-H_6_ at δ_C_/δ_H_ 119.4/6.82 ppm, respectively. In contrast to the spectrum of CL, some correlations, including the correlations at δ_C_/δ_H_ 106.2/6.51 and 112.7/6.77, which corresponded to the condensed S_2,6_ and G_2_ units [30], were observed in the spectra of CL and DPL. These results confirmed that the DAW process could not improve the removal of condensed units from CL. However, the cross-signals of these condensed units were very weak in PPL, which indicated that the PAW process was more selective in removing the condensed units from CL.

The quantitative analysis of the main linkages in the lignin samples was performed on the basis of previous 2D-HSQC methods [26]. As shown in Figure 2, the content of β-O-4 linkages in CL was 26.3/100Ar, which increased to 27.6/100Ar in DPL and 30.7/100Ar in PPL. This suggests that the β-O-4 linkages were enriched during the PAW process. It was previously reported that some condensed C–C linkages would be formed during ethanol organosolv pulping, such as β–β and β-5 bonds. In contrast to the C–C linkages (12.7/100Ar β–β and 8.5/100Ar β-5 linkages) in CL, the contents of C–C linkages in DPL and PPL were reduced. These results confirmed that the condensed lignin units were removed during the DAW and PAW processes. The changes in the S/G ratio among CL, DPL and PPL samples were also evaluated. It can be seen that the S/G ratio in CL (1.8) and DPL (1.8) was consistent, which indicated that the DAW process had little impact on the S/G ratio. However, the S/G ratio in PPL was increased compared to that in CL. These results indicated that the S-type lignin was enriched during the PAW process.

### 3.3. Indentification and Quantification of Functional Groups

**Figure 3:**
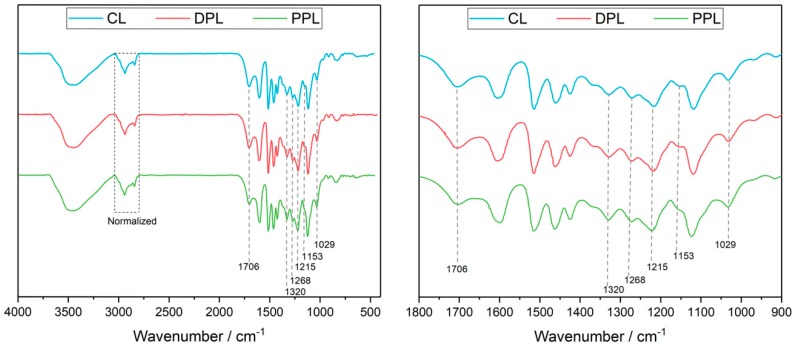
Fourier transform infrared spectroscopy (FTIR) spectra of CL, DPL and PPL.

The functional groups within lignin fractionations were generally characterized by FTIR. The spectra obtained from CL, DPL and PPL are shown in Figure 3. The wide band observed around 3334 cm^−1^ was the absorption signal of the O–H stretching vibration, which was ascribed to the OH groups in both phenolic and aliphatic structures. The bands at 2940 and 2838 cm^−1^, which correspond to the C–H vibration sketch in the CH_2_ and CH_3_ groups, respectively, were used to normalize the two spectra. The bands at 1706 cm^−1^ represented the C=O bonds in non-conjugated ketones. The intensity differences of the lignin bonds observed in the three spectra reflected the fact the PPL contains fewer non-conjugated ketone structures, while the CL and DPL have similar content. It was confirmed that the dioxane–acidic water process had a better selectivity for the non-conjugated ketones compared to the pyridine–acetic acid–water process. The bands at 1320 cm^−1^ (aromatic ring breathing, S and G condensed units), 1268 cm^−1^ (guaiacyl ring breathing with C=O stretching) and 1215 cm^−1^ (ring breathing with C–C, C–O and C=O stretching) were also present. Moreover, the bands at 1115 and 1029 cm^−1^ arose from the aromatic rings in the plane C–H bending deformation for S- and G-type lignin, respectively. The remaining sugar contents of the lignin-rich residues were also visible in the three spectra, which were revealed by the difference in the intensity bands at 1153 cm^−1^. The PPL was shown to have the lowest sugar contents compared to CL and DPL.

**Figure 4:**
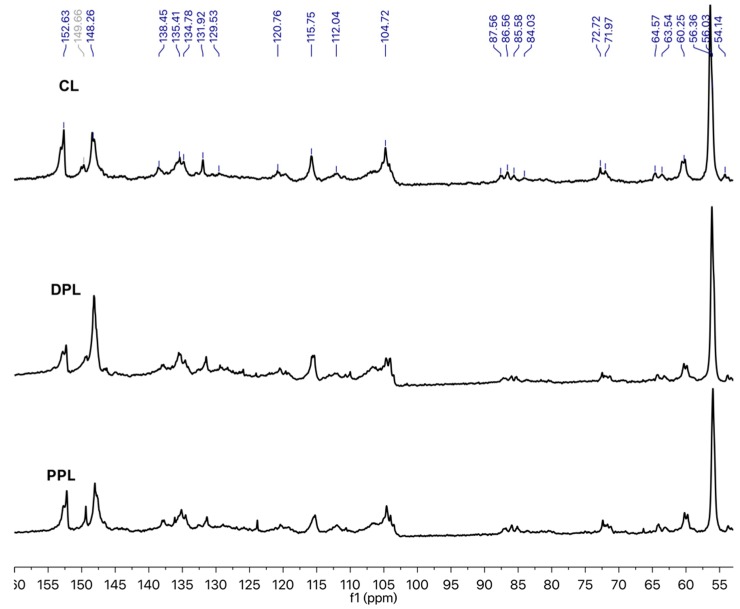
Quantitative ^13^C NMR spectra of CL, DPL and PPL.

The ^13^C NMR spectra of the three lignin preparations are depicted in Figure 4. The signals in the ^13^C NMR spectra were carefully assigned according to previous literature [23,26]. In the aromatic region between 102 and 160 ppm, the common signals that are associated with lignin-forming units, i.e., syringyl (S), guaiacyl (G) and *p*-hydroxybenzoate (PB), were observed. The S units resulted in signals around 152, 148, 138, 135 and 104 ppm. The typical chemical shifts that are related to G units were found at 148 and 120 ppm, while the presence of PB units was reflected by the signal at 115 ppm. Most of the signals between 50 and 102 ppm are related to the polysaccharide contamination and lignin interunit linkages. The β, α and γ carbons in the β-O-4 linkages were found around 85, 72 and 60 ppm, respectively. The presence of the methoxyl groups was verified by the intense signal found at 54–58 ppm.

In order to better evaluate the variation in the aromatic/aliphatic structures of the obtained lignin samples and the quantity of certain functional groups and structural units, the relative abundance (integral) of several groups of signals was determined by using the integral of the aromatic region of 102–162 ppm as a reference (assuming that it contains six aromatic carbons) [31,32]. The regions whose intensities were evaluated were those related to aromatic C–H (δ 123.0–103.0 ppm), aromatic C–C (δ 140.0–123.0 ppm), aromatic C–O (δ 160.0–140.0 ppm), β-O-4 (δ 61.0–58.0 ppm), Alkyl-O (δ 91.0–58.0 ppm) and methoxyl (OCH_3_) (δ 58.0–54.0 ppm). Table 2 provides the major signal assignments and the quantification results of the signals for CL, DPL and PPL. The proportion of Alkyl-O found in the DPL and PPL was lower compared with the CL Alkyl-O amount. On the other hand, the proportion of aromatic C–O was relatively higher in the DPL and PPL compared to that in CL. Both results could indicate a higher phenolic content in DPL and PPL than in CL. The decrease in the aromatic C–C, aromatic C–H and aliphatic structures were found in DPL and PPL, which suggests that the small molecular weight of the aliphatic structures found in the enriched lignin fractions was due to the DAW and PAW processes. Meanwhile, the contents of the OCH_3_ in the DPL and PPL samples were 0.90/Ar and 1.55/Ar and both of them were higher than those in CL (0.73/Ar). This indicated that the S-type lignin was more likely to be enriched during the PAW process compared to the DAW process, which was also confirmed by the 2D-HSQC NMR spectra. Additionally, the degree of condensation (DC) was determined by subtracting the observed signal intensity for C_Ar–H_ (δ 123–103 ppm) from the theoretical value of C_Ar–H_ [32]. The DC values of CL, DPL and PPL were 36%, 18% and 16%, respectively. These results confirmed that the condensed lignin units were removed during DAW and PAW processes, which was also confirmed by the 2D-HSQC NMR spectra.

### 3.4. Molecular Weight Distributions

**Figure 5:**
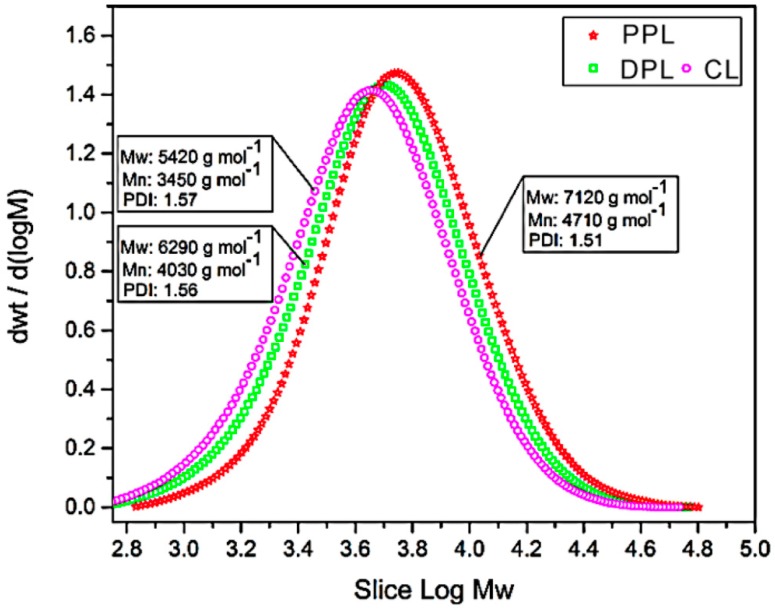
The GPC curves of CL, DPL and PPL samples.

The molar mass distribution curves of the CL, DPL and PPL samples are shown in Figure 5, in which their weight-average (M_W_) molecular weights, number-average (Mn) molecular weights and polydispersity indexes (M_W_/Mn, PDI) are also summarized. The M_W_ of CL was 5420 g mol^−1^, which was increased by 16% to 6290 g mol^−1^ in DPL and further increased by 31% to 7120 g mol^−1^ in PPL. It was deduced that the small molecular weight of the CL fractions was removed through the DAW and PAW processes, which were also confirmed by the increased amounts of β-O-4 linkages in the DPL and PPL samples as determined by 2D-HSQC NMR. Apart from the molecular weight, the PDI was also found to be reduced from 1.57 in CL to 1.56 in DPL and 1.51 in PPL, respectively, which suggests that the structures of DPL and PPL were more homogeneous than those of CL. Furthermore, the PPL exhibited relatively higher molecular weights compared to DPL, which suggests that the higher molecular weight lignin fractions were more easily obtained through the PAW process compared to the DAW process. Hence, the PPL with higher molecular weights that were obtained in this study would be suitable as a raw material in the synthesis of lignin-based carbon fibers [33].

### 3.5. Thermal Stability

**Figure 6:**
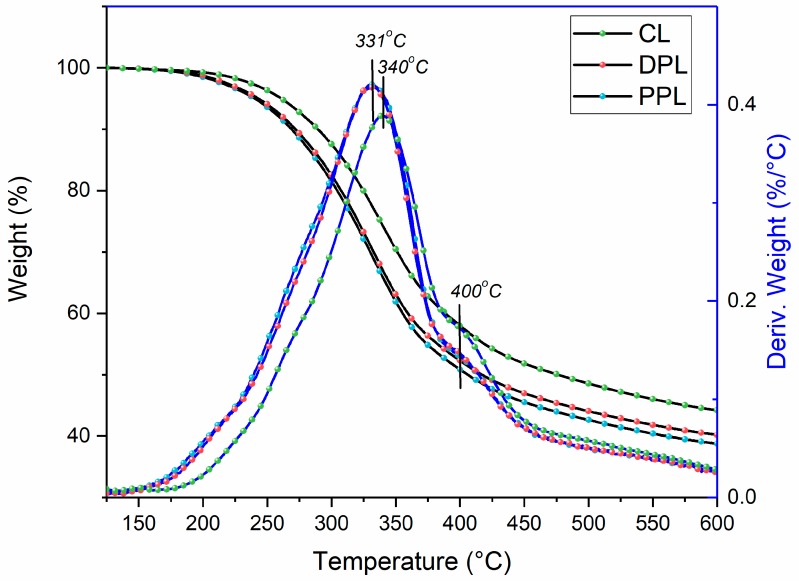
Thermogravimetric curves and derivative thermogravimetry curves of CL, DPL and PPL.

The thermal properties of lignin are extremely important for its utilization in the thermo-chemical conversion into energy and chemicals, such as thermolysis [6]. It is important to investigate the potential relationship between the quantitative structures and thermolysis. Once the relationship is established, it is possible to forecast the yield of pyrolysis products based on the quantitative structure of lignin polymers. The thermogravimetric weight loss curves (TGA) of the CL, DPL and PPL samples and their first derivative curves (DTG) are shown in Figure 6. Generally, the majority of aryl ether bond linkages were degraded at the temperature range of 200–350 °C. In this decomposing temperature range, the weight losses for DPL and PPL samples were separately 35.6% and 36.6%, both of which were higher than 28.8% for CL. These results indicated that the thermal stability of DPL and PPL was higher than that of CL during the initial decomposing phase, which were mainly attributed to their higher contents of β-O-4 linkages. This was also confirmed by the ^13^C NMR and 2D-HSQC NMR spectra.

The aromatic rings and C–C linkages (e.g., β-β and β-5 linkages) were decomposed when the temperature was above 400 °C. In the temperature range of 400–600 °C, the weight losses of DPL and PPL were 12.0% and 12.1%, which were lower than that of CL (13.8%). Meanwhile, the first derivative curve of CL was more obvious at 400 °C. These results indicated that a greater amount of resistant β-β and β-5 linkages was removed during the DAW and PAW processes. Meanwhile, it was found that the weight values of char residues at 600 °C were 44.2% in CL, 40.0% in DPL and 38.7% in PPL, which indicates that more condensed lignin structures were removed during the DAW and PAW processes. This was also confirmed by the aforementioned results in the ^13^C NMR and 2D-HSQC NMR spectra.

In addition, the maximum decomposition temperatures (T*m*) of lignin were observed to be reduced from 340 °C in CL to 331 °C in DPL and PPL. The T*m* of lignin samples shifted to lower temperatures after the DAW and PAW processes, which suggests that less stable lignin structures, such as condensed lignin structures, were removed during the DAW and PAW processes.

## 4. Conclusions

The structural recognition of the poplar organosolv lignin samples during the DAW and PAW purification processes were extensively investigated in this present study. Our results indicated that the PPL fractions were shown to contain fewer condensed structures, higher S/G ratio, more β-O-4 linkages, higher M_W_ and lower thermal degradation properties compared to the crude lignin and DPL fractions. Meanwhile, the PAW process was more selective for removing the condensed units and enriching S-type lignin from CL compared to the DAW process, which was indicated by the results of the quantitative ^13^C NMR, 2D-HSQC NMR spectra and thermogravimetric analysis. Based on the results, the purification of lignin in different stages should be carefully selected to tailor the lignin properties to those required for the final application.

## Figures and Tables

**Table 1:** The yields and chemical compositions of crude lignin (CL), DPL and PPL.

Samples	Yields (wt %) ^1^	Sugar Compositions (wt %) ^2^						Ash (wt %)
		Ara	Gal	Glu	Xyl	Man	Total	
CL	-	0.02	0.07	0.20	0.92	0.9	2.11	1.34
DPL	77.3	0.01	0.03	0.11	0.63	0.04	0.82	0.37
PPL	43.9	ND ^3^	ND	0.01	0.2	ND	0.21	ND

^1^ yield = DPL or PPL/CL × 100%. ^2^ Ara, arabinose; Gal, galactose; Glu, glucose; Xyl, xylose; Man, mannose. ^3^ Not detectable.

**Table 2:** The assignments and quantitative results of main chemical shifts in ^13^C NMR spectra of CL, DPL and PPL samples.

δ (ppm)	Assignments	Quantitative Results (/Ar)		
		CL	DPL	PPL
140–160	Aromatic C–O	2.20	2.39	2.53
123–140	Aromatic C–C	2.02	1.90	1.82
103–123	Aromatic C–H	2.03	1.69	1.75
58–91	Alkyl–O	2.83	1.21	1.01
58–61	β-O-4	0.28	0.38	0.42
54–58	OCH_3_	0.73	0.90	1.55
Degree of condensation		0.36	0.18	0.16
